# Protective effect of mesenchymal stem cells on the pressure ulcer formation by the regulation of oxidative and endoplasmic reticulum stress

**DOI:** 10.1038/s41598-017-17630-5

**Published:** 2017-12-07

**Authors:** Sei-ichiro Motegi, Akiko Sekiguchi, Akihiko Uchiyama, Akihito Uehara, Chisako Fujiwara, Sahori Yamazaki, Buddhini Perera, Hideharu Nakamura, Sachiko Ogino, Yoko Yokoyama, Ryoko Akai, Takao Iwawaki, Osamu Ishikawa

**Affiliations:** 1 Department of Dermatology, Gunma University Graduate School of Medicine, Maebashi, Japan; 2 Division of Plastic Surgery, Gunma University Graduate School of Medicine, Maebashi, Japan; 3 Division of Cell Medicine, Department of Life Science, Medical Research Institute, Kanazawa Medical University, Ishikawa, Japan

## Abstract

Cutaneous ischemia-reperfusion (I/R) injury is associated with the early pathogenesis of cutaneous pressure ulcers (PUs). The objective of this study was to investigate the effect of mesenchymal stem cells (MSCs) injection on the formation of PUs after I/R injury and determine the underlying mechanisms. We found that the subcutaneous injection of MSCs into areas of I/R injured skin significantly suppressed the formation of PUs. I/R-induced vascular damage, hypoxia, oxidative DNA damage, and apoptosis were decreased by MSCs injection. Oxidative stress signals detected after I/R in OKD48 (Keap1-dependent oxidative stress detector, No-48-luciferase) mice were decreased by the injection of MSCs. In cultured fibroblasts, MSCs-conditioned medium significantly inhibited oxidant-induced reactive oxygen species (ROS) generation and apoptosis. Furthermore, endoplasmic reticulum (ER) stress signals detected after I/R in ERAI (ER stress-activated indicator) mice were also decreased by the injection of MSCs. These results suggest that the injection of MSCs might protect against the development of PUs after cutaneous I/R injury by reducing vascular damage, oxidative cellular damage, oxidative stress, ER stress, and apoptosis.

## Introduction

Because of population aging, the number of patients with pressure ulcers (PUs) is increasing worldwide. PUs are a significant cause of pain and distress, leading to an impaired quality of life^1^. Therefore, the prevention of PUs is an important issue. In the early stage of PUs formation, non-blanchable erythema and/or purpuric lesions appear at an area of the skin subjected to physical pressure and a skin ulcer develops 2 or 3 weeks later. Many evidence-based treatments have been developed for the treatment of established skin ulcers^2,3^. However, there is currently no evidence-based early-stage treatment to prevent the development of skin ulcers. Therefore, identifying appropriate treatments or techniques to prevent the development of skin ulcers from the initial stage of non-blanchable erythema is essential.

Tissue damage due to pressure-induced ischemia-reperfusion (I/R) injury, which is defined as cellular damage caused by the reperfusion of blood into ischemic tissue, has recently been considered to be associated with the pathogenesis of PUs^4–10^. Using a simple, non-invasive, and clinically relevant mouse model of PUs, we and other groups have demonstrated that cutaneous I/R injury can induce pathogenic events, such as damage to endothelial cells (ECs), thrombus, edema, production of proinflammatory cytokines from infiltrated leukocytes and macrophages, and thereafter, apoptosis and necrosis in the tissue^6–10^. In addition, we previously demonstrated that the inhibition of I/R injury-induced oxidative stress in the acute phase protected against the subsequent formation of skin ulcers^8–10^.

Mesenchymal stem cells (MSCs) are bone marrow-derived, non-hematopoietic progenitor cells. MSCs can differentiate into various cell types such as chondrocytes, adipocytes, osteocytes, myocytes, ECs and keratinocytes^11,12^. Previous studies have reported that the intravenous or intradermal administration of MSCs promotes cutaneous wound healing in animals and humans^12–15^. Several mechanisms have been identified for promoting wound healing by MSCs, including the enhancement of angiogenesis via the secretion of pro-angiogenic factors, differentiation of MSCs into endothelial cells, pericytes, fibroblasts, and keratinocytes, promotion of M2 macrophages infiltration, recruitment of endogenous stem/progenitor cells, extracellular matrix production and remodeling, and immunosuppressive effects^12,15–19^.

In addition, many studies have suggested that MSCs prevent I/R injury in various organs, including the heart^20^, lung^21^, kidney^22^, and liver^23^. However, there have been no reports demonstrating the effect of MSCs on cutaneous I/R injury and the subsequent formation of skin ulcers. Therefore, in this study, we examined the effect of MSCs on the acute phase of PUs formation after cutaneous I/R and investigated the underlying mechanisms.

## Results

### Injected MSCs protected against PUs formation in a mouse model of cutaneous I/R injury

**Figure 1:**
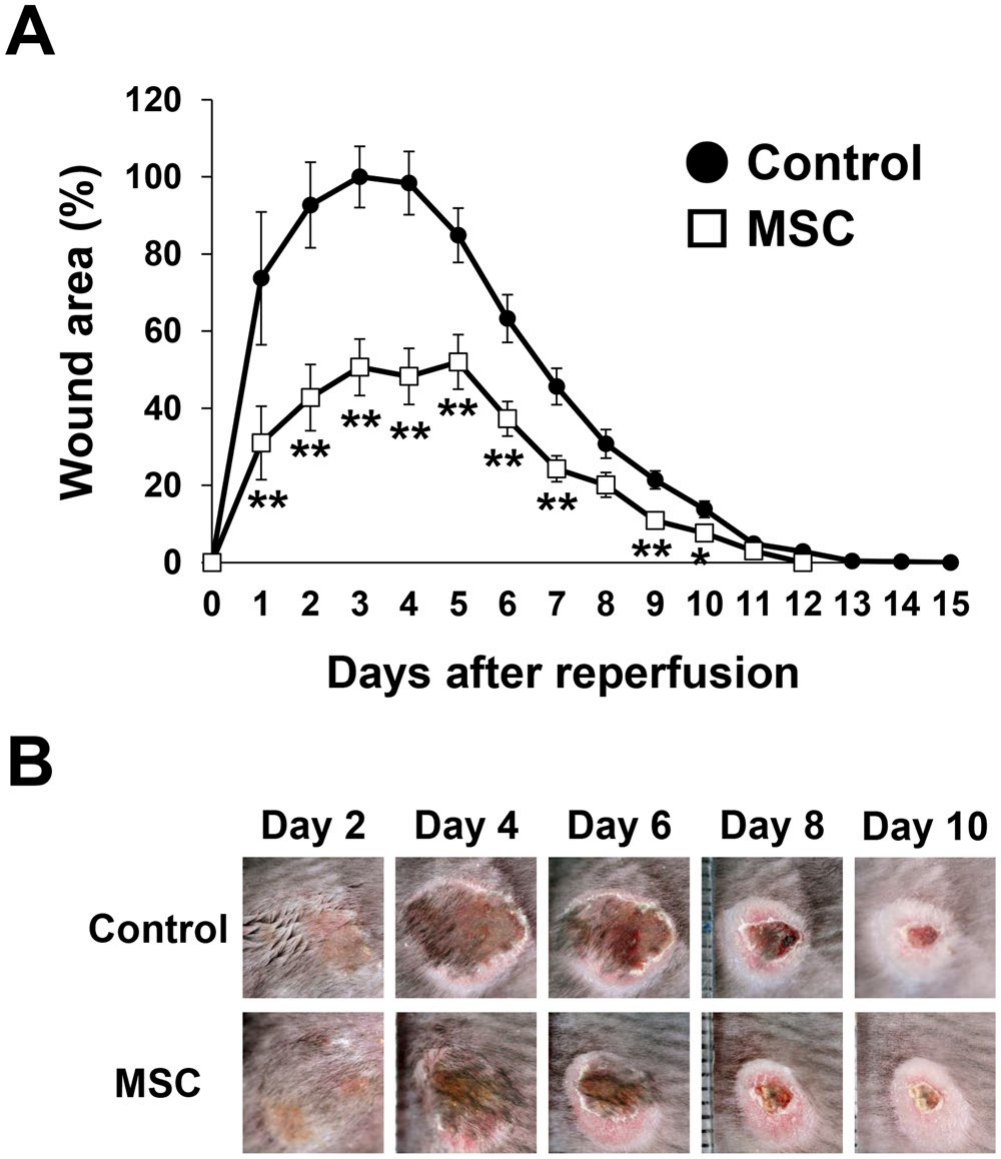
Injected MSCs protected against PUs formation in a mouse model of cutaneous I/R injury. (**A**) Percent wound area at each time point relative to the wound area in control mice at 3 days after reperfusion (n = 14 for each time point and groups). All values represent mean ± SEM. **P < 0.01, *P < 0.05. (**B**) Photographs of wound after cutaneous I/R in control or MSCs injected mice at 2, 4, 6, 8 and 10 days after reperfusion.

At first, we examined the effects of MSCs on the development of PUs after cutaneous I/R injury *in vivo*. Either MSCs or phosphate-buffered saline as a control were subcutaneously injected into the dermis at the I/R site immediately after reperfusion (at Day 0) and the wound area was compared between the groups at several time points. Several reports have found that the hypoxic preconditioning of MSCs before their implantation into tissues enhanced the survival of the implanted MSCs and angiogenesis in the treated tissues, resulting in the accelerated repair of damaged tissues such as diabetic intractable ulcers and infarcted myocardium^15,24^. Therefore, MSCs were incubated under hypoxic conditions for 24 hours prior to implantation into the dermis in this study. The administration of MSCs significantly suppressed the formation of cutaneous PUs after I/R injury (Fig. 1A,B). At 3 days after reperfusion, the size of the wound area in the MSCs-injected mice was 50% of that in the control mice. The wound area in the MSCs-injected mice was significantly smaller than that in control mice from 1 to 10 days after reperfusion. The wound closure time in control mice was longer than that in MSCs-injected mice (12.8 vs. 11.6 days, *P* < 0.01). These results suggested that MSCs might protect against the formation of PUs after cutaneous I/R injury.

### Injection of MSCs prevented the depletion of blood vessels by cutaneous I/R injury

**Figure 2:**
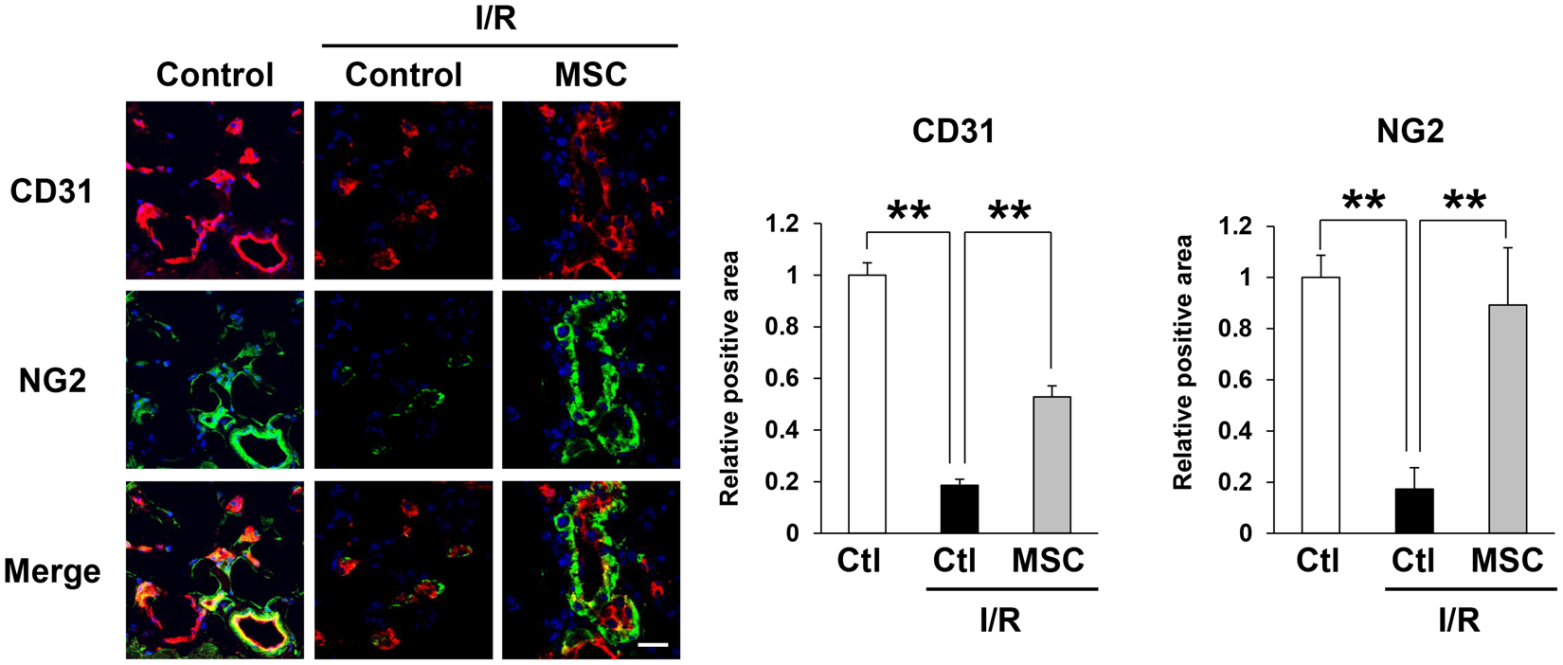
Injection of MSCs prevented the depletion of blood vessels by cutaneous I/R injury. The amount of CD31^+^ EC and NG2^+^ pericytes in cutaneous I/R area at 4 days after reperfusion. Quantification of the CD31^+^ and NG2^+^ areas in 6 random microscopic fields from the periphery of I/R area in n = 3 mice per groups was performed using Image J software. Positive area in control mice was assigned a value of 1. Values represent mean ± SEM. **P < 0.01. Scale bar = 20 μm.

We previously determined that the number of blood vessels was reduced after cutaneous I/R injury in a mouse model^8^. Therefore, we next investigated the effect of MSCs on the cutaneous I/R injury-induced reduction of vascularity. At 4 days after reperfusion, the numbers of CD31^+^ ECs and NG2^+^ pericytes in the I/R-injured areas were significantly reduced in comparison with those in control mice without I/R injury treatment, while MSCs injection protected against the reduction in the numbers of both ECs and pericytes (Fig. 2). These results suggested that MSCs might prevent cutaneous I/R injury-induced vascular damage.

### Injection of MSCs reduced the induction of hypoxia, oxidative stress, and apoptosis by cutaneous I/R injury

**Figure 3:**
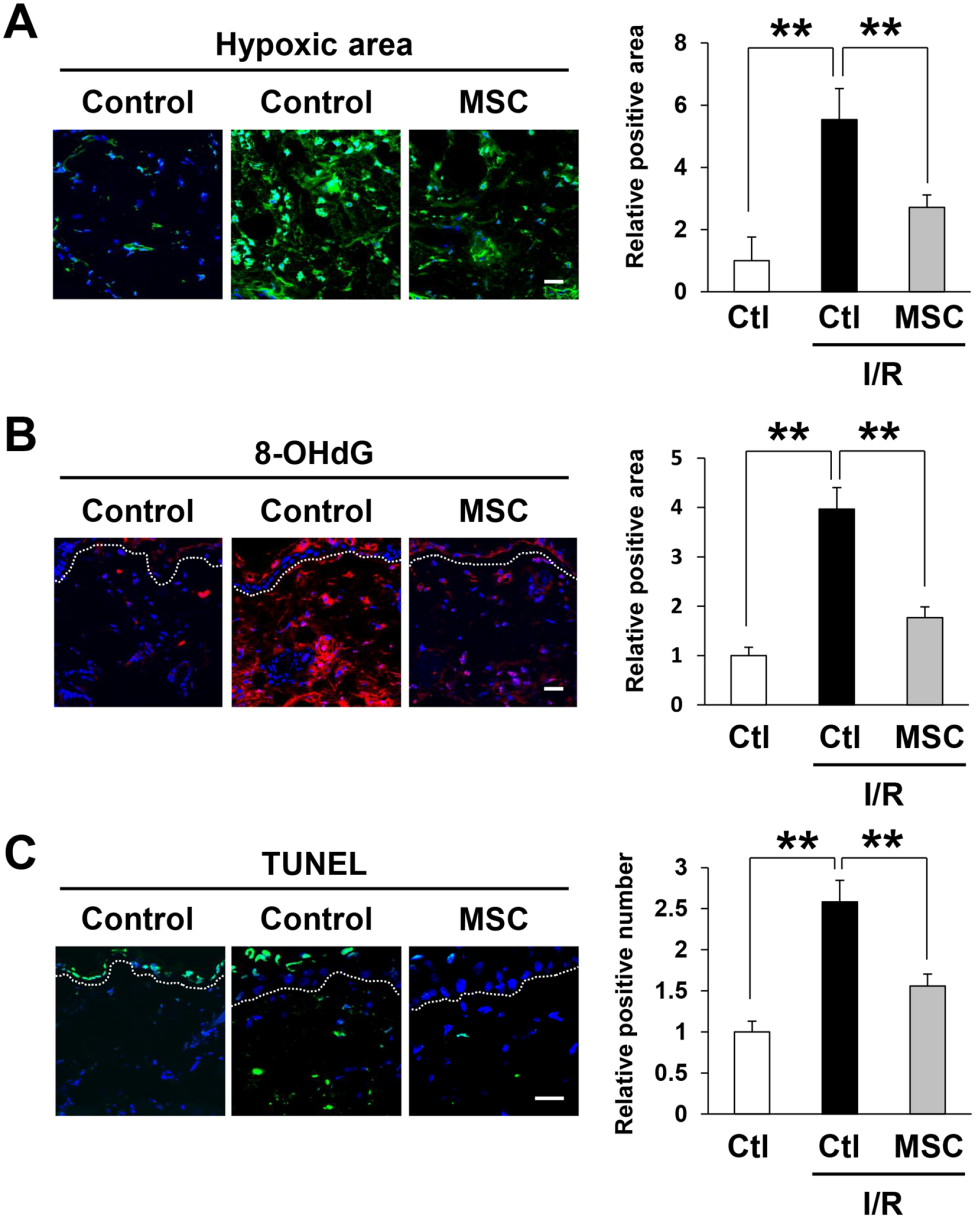
Injection of MSCs reduced the induction of hypoxia, oxidative stress, and apoptosis by cutaneous I/R injury. (**A**) The amount of pimonidazole^+^ hypoxic area (green) in cutaneous I/R site at 1 day after reperfusion. (**B**) The amount of 8-OHdG^+^ DNA damaged area (red) in cutaneous I/R site at 1 day after reperfusion. Quantification of the 8-OHdG^+^ areas and pimonidazole^+^ areas in 6 random microscopic fields from the center of I/R area in n = 3 mice per groups was performed using Image J software. Positive area in control mice was assigned a value of 1. (**C**) The number of apoptotic cells in I/R site at 1 day after reperfusion was determined by counting both TUNEL and DAPI positive cells. Values were determined in 6 random microscopic fields from the center of I/R area in n = 3 mice per groups. The number of apoptotic cells in control mice was assigned a value of 1. Values represent mean ± SEM. **P < 0.01, *P < 0.05. Scale bar = 20 μm.

It has been reported that reactive oxygen species (ROS) are produced by cutaneous I/R injury and create 8-hydroxy-2′-deoxyguanosine (8-OHdG), which is a specific marker of oxidative stress-associated DNA damage, in tissue-resident cells^7,9^. To examine the effects of MSCs on hypoxia and oxidative damage after I/R injury in mice, immunofluorescence staining was performed on skin tissue sections from the area subjected to I/R, using an anti-pimonidazole antibody, which specifically recognizes hypoxic cells, and an anti-8-OHdG antibody, respectively. At one day after reperfusion, the hypoxic area (Fig. 3A) and oxidatively damaged area (Fig. 3B) in the I/R-injured areas were significantly increased in comparison with those in control mice without I/R injury treatment, while MSCs injection significantly reduced I/R-induced hypoxic area and oxidatively damaged area (Fig. 3A,B). We also examined the effects of MSCs on the number of apoptotic cells after I/R in mice. At one day after reperfusion, the numbers of TUNEL^+^ apoptotic cells in the I/R areas were significantly higher than those in control mice without I/R injury treatment, while MSCs injection significantly inhibited I/R-induced apoptotic cells in I/R area (Fig. 3C). Furthermore, we examined the hypoxic area, oxidatively damaged area and the numbers of apoptotic cells in the I/R areas 4 days after reperfusion. Similar to the results of one day after reperfusion, I/R-induced hypoxic area, oxidatively damaged area and the numbers of apoptotic cells were significantly suppressed by MSCs injection (Supplemental Figure S1A–C). These results suggested that MSCs might mitigate the hypoxic area, oxidative stress, and apoptosis induced by cutaneous I/R injury.

### Injection of MSCs reduced oxidative stress induced by cutaneous I/R injury *in vivo*

**Figure 4:**
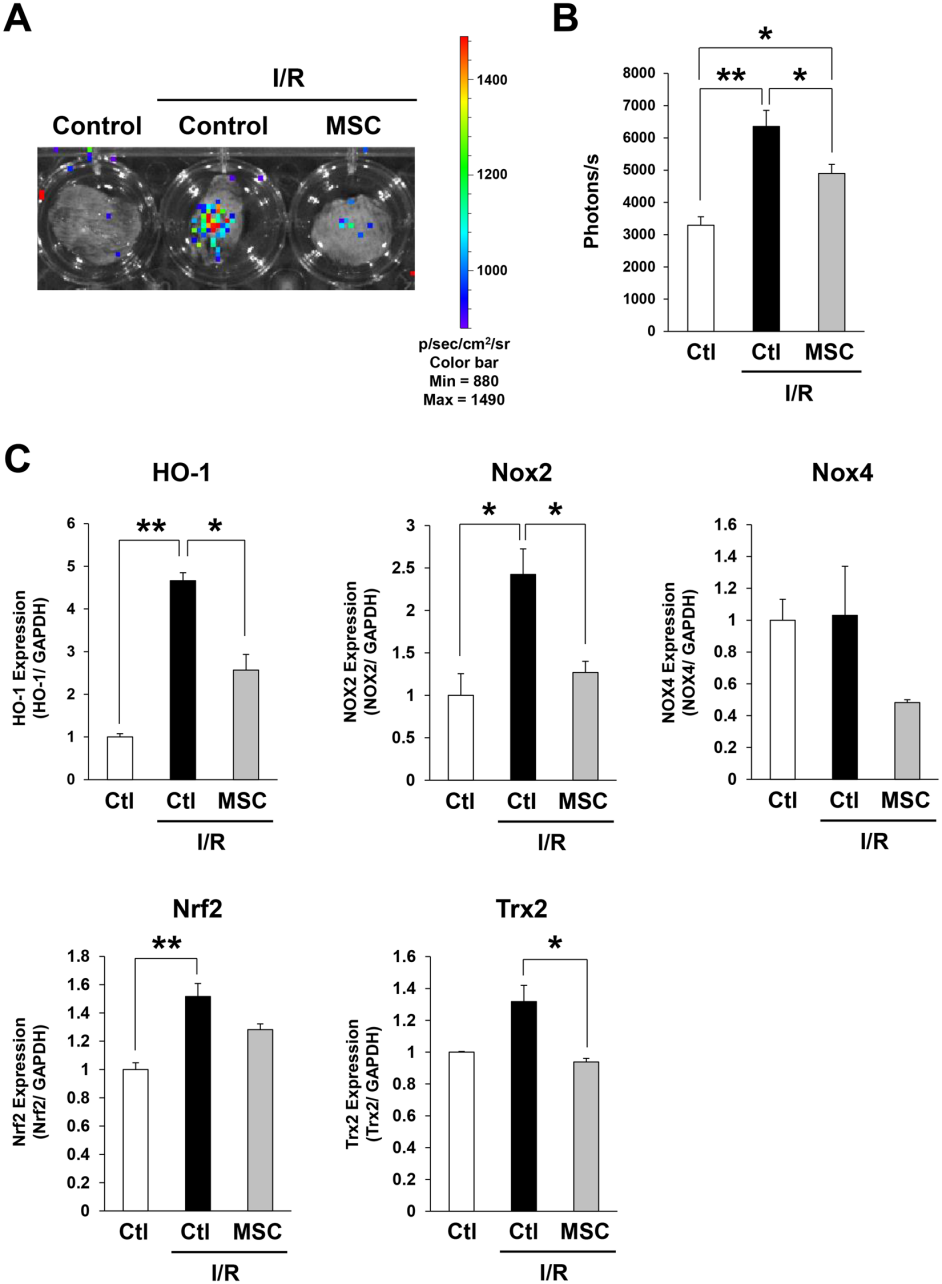
Injection of MSCs reduced oxidative stress induced by cutaneous I/R injury *in vivo*. (**A**) Representative image of luminescence signals in cutaneous I/R area in OKD 48 mice at 1 day after reperfusion. The color scale bar shows the photon counts (photon(p)/sec/cm2/sr). (**B**) Quantification of luminescence signals in cutaneous I/R area in OKD 48 mice. Values represent mean ± SEM (n = 7–8 in each group). **P < 0.01, *P < 0.05. (**C**) mRNA levels of oxidative stress-associated factors, HO-1, Nox2, Nox4, Nrf2 and Trx2 in the I/R area at 1 day after reperfusion. mRNA levels in control mice were assigned values of 1. Values represent mean ± SEM (n = 3–8 in each group). **P < 0.01, *P < 0.05.

We further investigated oxidative stress induced by I/R injury using OKD48 (Keap-1 dependent oxidative stress detector, No-48) mice^25^. OKD48 mice have a transgene encoding a modified nuclear factor (erythroid-derived 2)-related factor 2 (Nrf2) protein, which is an essential transcription factor for the expression of anti-oxidative stress genes^26^. Using this strain of mice, oxidative stress can be detected *in vivo* by luminescence signals^25,27^. At one day after reperfusion, a strong luminescence signal was detected in the area subjected to I/R, and this signal was significantly suppressed by the injection of MSCs (Fig. 4A,B). In addition, by real-time PCR, we examined the mRNA levels of oxidative stress-associated factors, including heme oxygenase 1 (HO-1), NADPH oxidases (Nox2 and Nox4), Nrf2, and thioredoxin 2 (Trx2) after I/R. *Hmox1* encodes HO-1, which is an important anti-oxidant enzyme^28^. NOX2 and NOX4 are essential enzymes for ROS production^29^. Nrf2 and Trx2 are essential for protection against oxidant-induced apoptosis^30,31^. It has been reported that the expression of HO-1, Nox, Trx2 and Nrf2 is enhanced by I/R injury in the cerebrum and liver^32–34^. We found that cutaneous I/R injury significantly increased the mRNA levels of HO-1, Nox2 and Nrf2 (Fig. 4C). However, the injection of MSCs reduced the I/R-induced mRNA levels of HO-1, Nox2 and Trx2 (Fig. 4C). These results suggested that the injection of MSCs might inhibit oxidative stress in a mouse model of cutaneous I/R injury.

### MSCs-conditioned medium suppressed the oxidant-induced intracellular accumulation of ROS and cell death in fibroblasts *in vitro*

**Figure 5:**
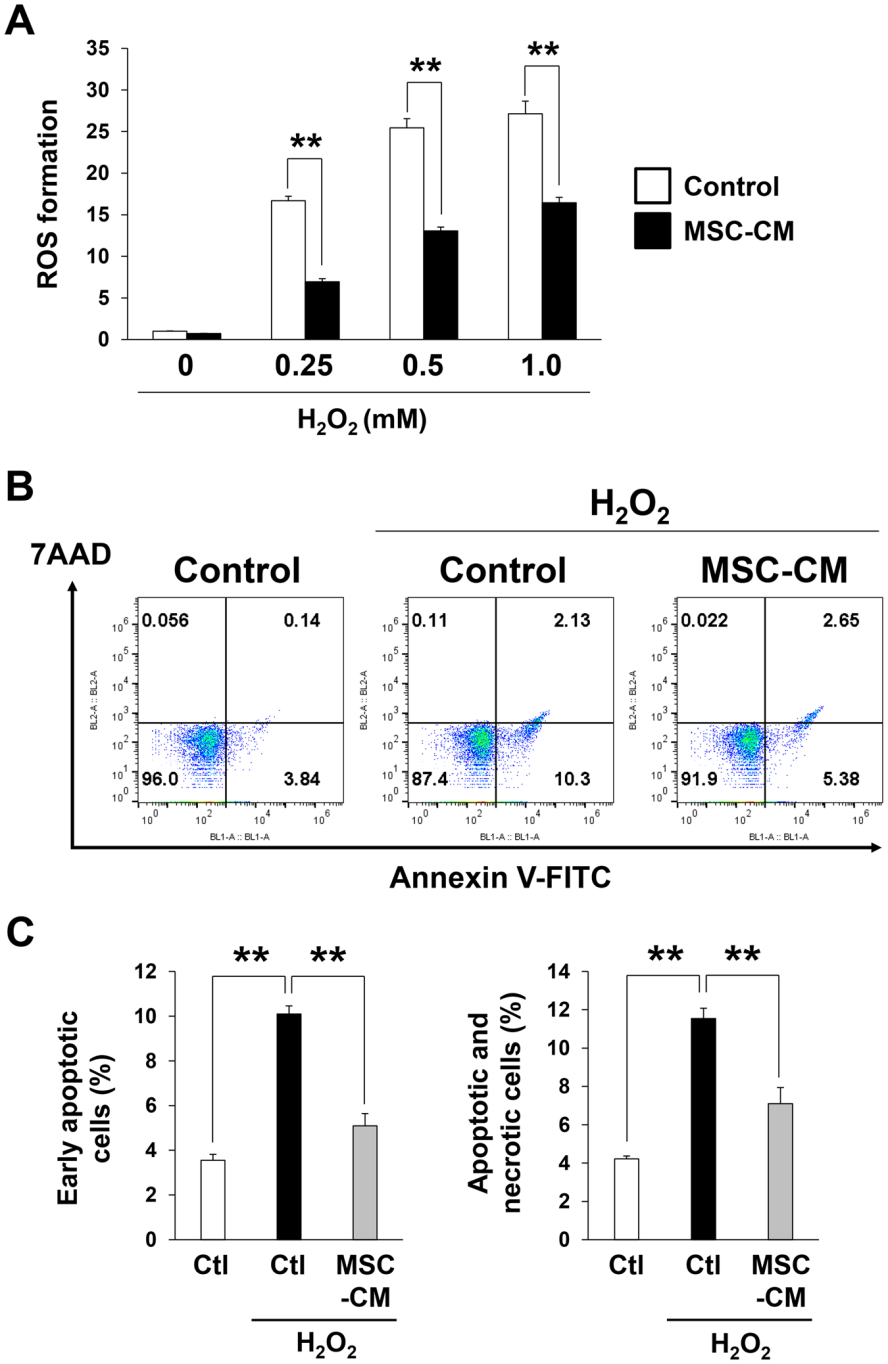
MSCs-conditioned medium inhibited the oxidant-induced intracellular accumulation of ROS and cell death in fibroblasts *in vitro*. (**A**) Quantification of H_2_O_2_-induced intracellular ROS production in NIH3T3 fibroblasts treated with control or MSCs-conditioned medium (MSCs-CM). ROS formation in cells with control medium treatment was assigned a value of 1. Values represent mean ± SEM. n = 4 in each group. **P < 0.01. (**B**) Representative data of the amount of early apoptotic cells (Annexin V^+^, 7-AAD^−^) and the total apoptotic and necrotic cells (Annexin V^+^) in NIH3T3 fibroblasts with or without H_2_O_2_ (0.5 mM) incubated with control or MSCs-CM. (**C**) Quantitation of the amount of early apoptotic cells (Annexin V^+^, 7-AAD^−^) and the total apoptotic and necrotic cells (Annexin V^+^) in NIH3T3 fibroblasts. Values represent means ± SEM in three independent experiments. **P < 0.01.

To examine the effect of MSCs on oxidative stress *in vitro*, we next examined the effect of MSCs-conditioned medium on the H_2_O_2_-induced accumulation of intracellular ROS in mouse fibroblasts (NIH3T3). The MSCs-conditioned medium suppressed ROS accumulation at various concentrations of H_2_O_2_ (Fig. 5A). Next, we examined the effects of MSCs-conditioned medium on H_2_O_2_-induced apoptosis and necrosis in NIH3T3 cells. The proportions of early apoptotic cells (annexin V^+^, 7-aminoactinomycin D (7-AAD)^−^) and total apoptotic and necrotic cells (annexin V+) were increased by H_2_O_2_ treatment (Fig. 5B,C). However, the H_2_O_2_-induced increases in the proportions of early apoptotic cells and total apoptotic and necrotic cells were significantly inhibited by the incubation with MSCs-conditioned medium (Fig. 5B,C). These results suggested that MSCs might reduce oxidative stress and oxidative stress-induced cell death *in vitro*.

### Injection of MSCs reduced endoplasmic reticulum (ER) stress induced by cutaneous I/R injury *in vivo*

**Figure 6:**
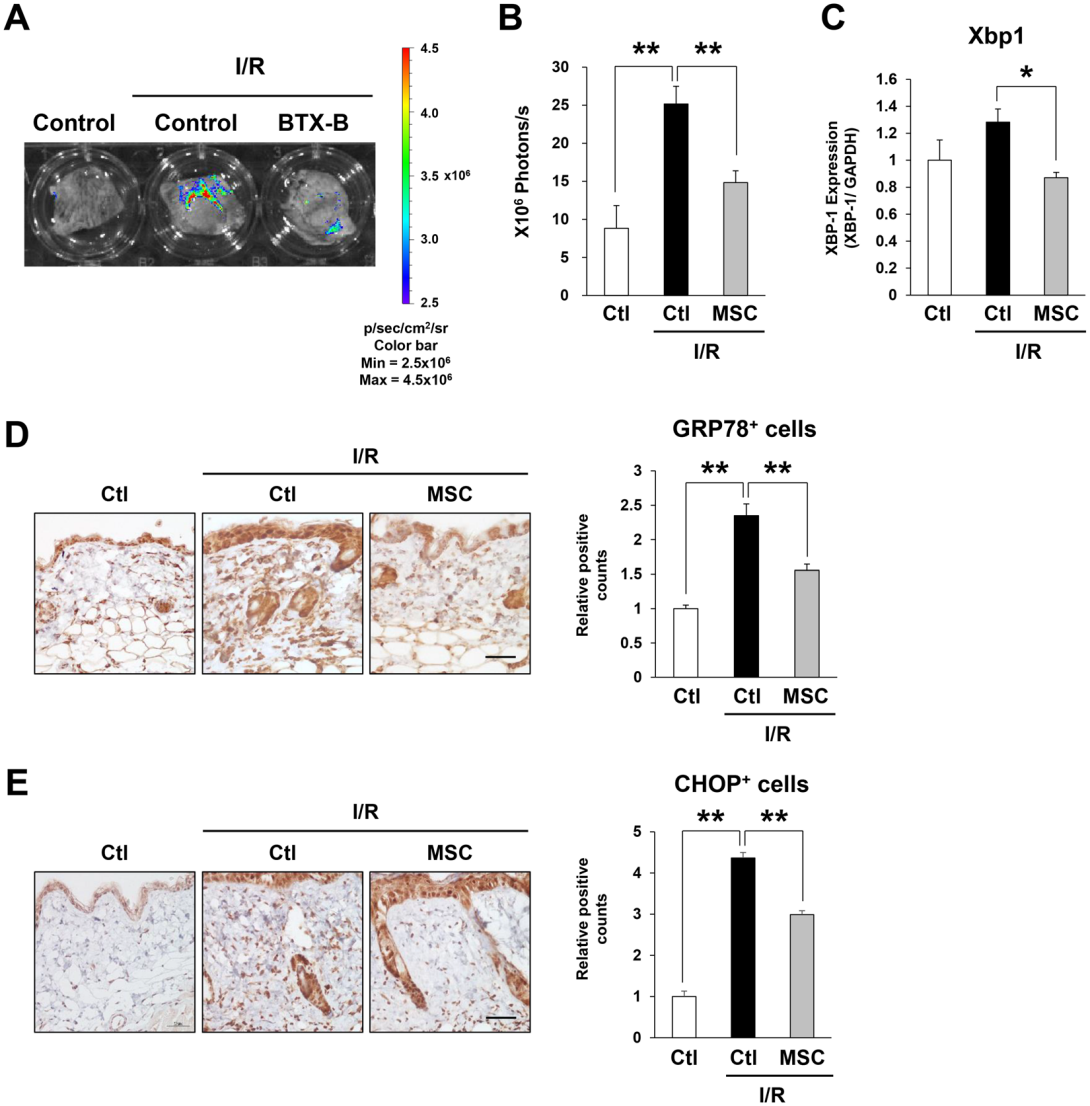
Injection of MSCs reduced endoplasmic reticulum (ER) stress induced by cutaneous I/R injury *in vivo*. (**A**) Representative image of luminescence signals in cutaneous I/R area in ERAI mice at 1 day after reperfusion. The color scale bar shows the photon counts (photon(p)/sec/cm2/sr). (**B**) Quantification of luminescence signals in cutaneous I/R area in ERAI mice. Values represent mean ± SEM. n = 4 in each group. **P < 0.01. (**C**) mRNA levels of Xbp1 in the I/R area at 1 day after reperfusion. mRNA levels in control mice were assigned values of 1. Values represent mean ± SEM (n = 3–4 in each group). *P < 0.05. (**D**,**E**) Quantification of GRP78/BiP positive cells (**D**) and CHOP positive cells (**E**) into I/R area at 1 day after reperfusion. The number of positive cells in control mice were assigned values of 1. Values represent mean ± SEM (n = 3–6 in each group). **P < 0.01.

It has been reported that hypoxia induces not only oxidative stress but also ER stress that was involved in the pathogenesis of I/R injury in the liver, heart, and kidney^22,35–37^. Therefore, we next analyzed the effect of MSCs injection on ER stress induced by cutaneous I/R injury. GRP78, which is also known as BiP, is a central regulator of ER stress^38^. When ER stress occurs, GRP78/BiP is released from ER transmembrane signal transducers, including PKR-like ER kinase (PERK), inositol-requiring enzyme 1 (IRE1), and activating transcription factor 6 (ATF6), leading to the activation of unfolded protein response (UPR) signaling pathways^38,39^. After dissociating from GRP78/BiP, IRE1 dimerizes to promote its autophosphorylation and activation. Activated IRE1 has an endoribonuclease activity and splices a 26-base intron from the mRNA encoding X-box binding protein 1 (XBP-1), which is an essential transcription factor for the expression of ER stress response genes^40^. In this study, we used ERAI (ER stress-activated indicator) transgenic mice, which have a transgene encoding a modified Xbp-1^41,42^. Using this strain of mice, ER stress can be detected *in vivo* by luminescence signals^41,42^. At one day after reperfusion, an ER stress signal was detected in the periphery of the area subjected to I/R (Fig. 6A,B). This signal was significantly suppressed by the injection of MSCs (Fig. 6A,B). Furthermore, we examined the effect of MSCs on ER stress-response factors. We found that the mRNA levels of Xbp1 in the cutaneous area subjected to I/R were significantly reduced by the injection of MSCs (Fig. 6C). In addition, the number of GRP78/BiP-positive cells, including fibroblasts, ECs, and infiltrating cells, in the dermis was increased by cutaneous I/R injury and significantly reduced by the injection of MSCs (Fig. 6D). C/EBP homologous protein (CHOP), which is also known as growth arrest- and DNA damage-inducible gene 153 (GADD153), is one of the downstream effectors of unfolded protein response signaling and induces mitochondria-dependent apoptosis^38,43^. Immunostaining for CHOP showed that cutaneous I/R injury increased the number of CHOP-positive cells, including fibroblasts, ECs, and infiltrating cells, in the dermis and this increase was significantly inhibited by the injection of MSCs (Fig. 6E). These results suggested that the injection of MSCs might reduce ER stress induced by cutaneous I/R injury *in vivo*.

## Discussion

We previously demonstrated that the injection of either recombinant MFG-E8, which is a secreted protein that acts as a ligand for integrins, or botulinum toxin A protected against the development of PUs after cutaneous I/R injury by regulating angiogenesis and suppressing hypoxia and oxidative stress-induced tissue damage^8,9^. We also determined that a topical steroid accelerated the formation of PUs induced by cutaneous I/R injury by enhancing oxidative stress-induced tissue damage^10^. This is the first study demonstrating that MSCs improved cutaneous I/R injury and protected against the development of skin PUs by inhibiting oxidative stress and ER stress during the acute phase of I/R injury. It has previously been reported that the intravenous or intradermal administration of MSCs improved I/R injury in various organs, including the heart^20^, lung^21^, kidney^22^, and liver^23^. However, the detailed mechanism by which the injection of MSCs improves I/R injuries remains to be fully elucidated. It has been considered that there are two main mechanisms underlying the acceleration of wound healing by MSCs: (I) their paracrine communication with resident cells in the wounds, such as infiltrating inflammatory cells, and antigen-presenting cells, through the release of cytokines, growth factors, and extracellular matrix proteins, and (II) their differentiation into resident cells^19,44^. These functions of MSCs may inhibit inflammation and enhance angiogenesis, granulation tissue formation, extracellular matrix remodeling and reepithelization in wounds. In a mouse model of cutaneous I/R injury, we and other groups previously found that damage to ECs, thrombus, edema, and the production of proinflammatory cytokines from infiltrated leukocytes and macrophages were induced in the early phase of I/R injury, leading to the apoptosis and necrosis in the damaged tissues and the development of PUs^6–10^. Therefore, transplanted MSCs need to work immediately after reperfusion to prevent various cellular dysfunctions and inflammatory cascades, suggesting that autocrine and paracrine communication via the secretion of growth factors and/or cytokines, rather than differentiation, might be more important in the early phase of I/R injury.

In this study, we found that the injection of MSCs prevented a reduction in the number of blood vessels after cutaneous I/R injury. It has been recognized that MSCs in the wound area secrete growth factors or cytokines such as vascular endothelial growth factor, platelet-derived growth factor, basic fibroblast growth factor (bFGF), and angiopoietin-1, resulting in the promotion of angiogenesis and wound healing^14,19^. In addition, we previously determined that green fluorescent protein-labeled MSCs injected into murine skin together with melanoma cells localized to the perivascular area in melanoma tumors and were still observed at 10 days after inoculation^17^. These results suggested that MSCs injected into cutaneous areas subjected to I/R might remain for several days and prevent thrombosis, rescue injured blood vessels, and promote angiogenesis by secreting growth factors and/or cytokines after cutaneous I/R injury.

A growing body of evidence suggests a critical role for oxidative stress in mediating tissue injury and cell death during I/R injury^45^. Free radical production after I/R is enhanced by the elimination of endogenous antioxidative systems in ischemic tissues, especially after reperfusion^45^. Elevated levels of ROS can directly disrupt the structures of lipids, proteins, and DNA and induce cell death in various pathways. Furthermore, ROS can serve as intracellular signaling molecules and control inflammation or the response to cellular injury. In this study, we confirmed that oxidative stress was elevated *in vivo* by cutaneous I/R and this elevation was suppressed by the injection of MSCs using OKD48 mice. In addition, the injection of MSCs suppressed the expression of Nox2 in skin after I/R. Nox2 is expressed mainly in macrophages and neutrophils but also in vascular ECs and fibroblasts^46^. Therefore, MSCs might suppress oxidative stress mainly in infiltrating macrophages and neutrophils but also in vascular ECs and fibroblasts. The mechanism by which MSCs suppress oxidative stress is still unknown. Jeon *et al*. reported that the incubation of cultured human fibroblasts with MSCs-conditioned medium enhanced the production of superoxide dismutase (SOD), which is an antioxidant enzyme^47^. In the present study, we demonstrated that MSCs-conditioned medium suppressed oxidant-induced ROS generation and apoptosis in cultured fibroblasts. These findings suggested that certain humoral factors derived from MSCs can directly suppress oxidative stress. However, the detailed mechanism is unknown and further investigation is warranted.

Hypoxia induces not only oxidative stress but also ER stress, and ER stress has been reported to be involved in the pathogenesis of I/R injury in the liver, heart, and kidney^22,35–37^. In addition, ROS is one of the key stimuli that can cause ER stress, and ER stress often accompanies an increase in ROS production^48^. Several studies have shown that MSCs can improve ER stress in animal models^22,49,50^. In animal models including renal injury induced by renal artery stenosis, spinal cord injury, and acute colitis induced by dextran sulfate sodium (DSS), the injection of MSCs significantly improved the injury or the disease activity and reduced ER stress and apoptosis^22,49,50^. In the present study, using ERAI mice to visualize ER stress, we found that ER stress was enhanced by cutaneous I/R injury at one day after reperfusion, and I/R-induced ER stress was suppressed by the injection of MSCs. Interestingly, the ER stress signal was strongly observed in the peripheral area of cutaneous I/R injury. Additionally, the numbers of GRP78/BiP-positive and CHOP-positive cells, including fibroblasts, ECs, and infiltrating cells, in the dermis were increased by cutaneous I/R injury and significantly decreased by the injection of MSCs. Since the ER stress signal was detected at one day after reperfusion and the signal gradually declined after the second day (data not shown), we suggest that cutaneous I/R-induced ER stress may play roles in the early phase of inflammation, and the attenuation of ER stress and apoptosis by MSCs contribute to the mitigation of tissue damage after cutaneous I/R injury. Although the suppressive mechanism of I/R-induced ER stress by MSCs is unknown, it is possible that ER stress was alleviated by MSCs via through the suppression of vascular dysfunctions and subsequent hypoxia.

**Figure 7:**
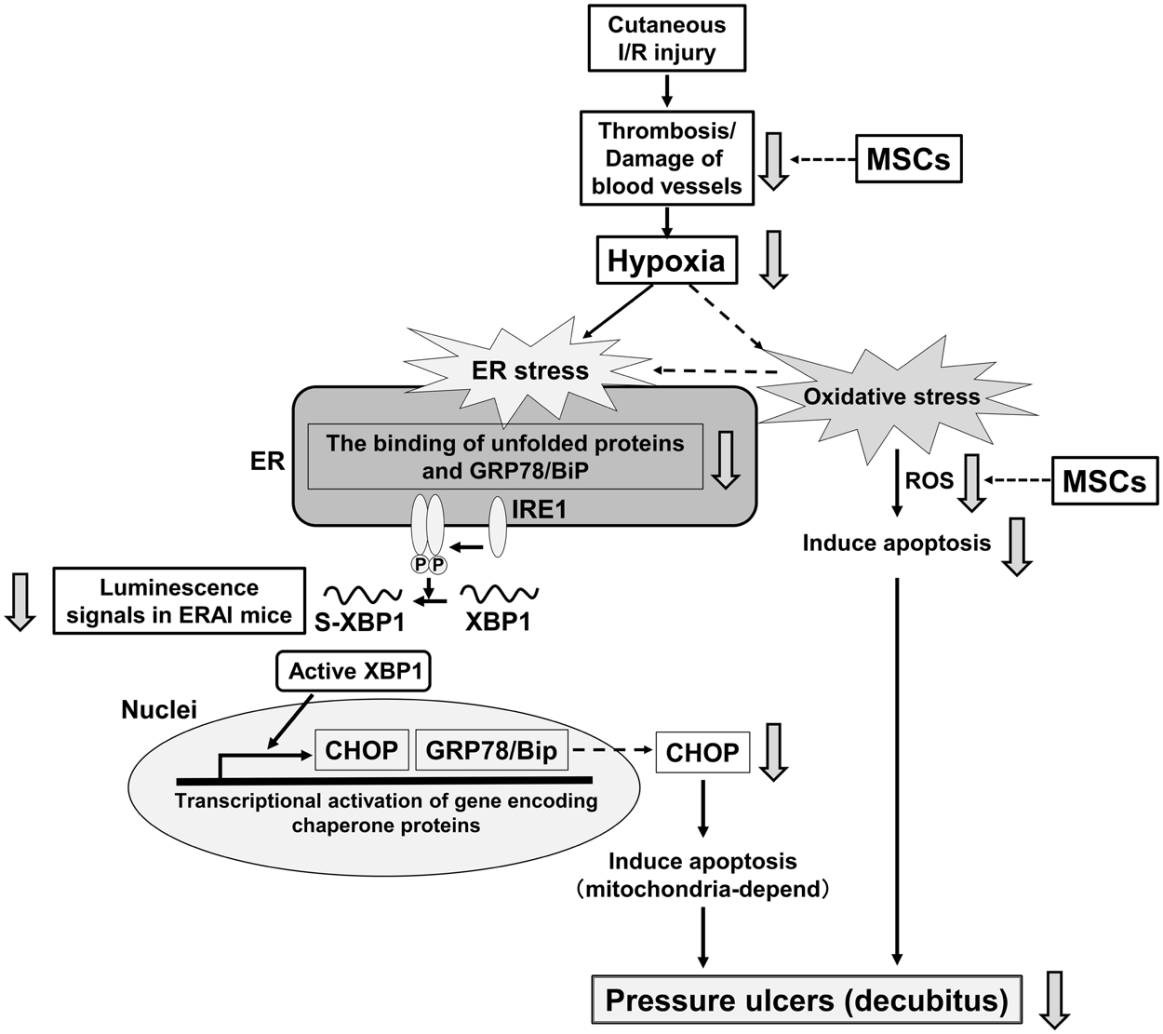
Model for the mechanism by which MSCs suppress the development of PUs in the mouse model of cutaneous I/R injury. Cutaneous I/R injury causes thrombosis and vascular injury, and subsequent tissue hypoxia. Hypoxia induces not only oxidative stress but also ER stress. The increased generation of ROS during oxidative stress causes apoptosis, while the increased expression of CHOP caused by ER stress may induce mitochondria-dependent apoptosis. These sequential responses may elicit PUs. The injection of MSCs may alleviate blood vessel damage and hypoxia, and subsequently suppress both oxidative stress and ER stress, resulting in the inhibition of PUs formation.

Based on the present our results, we propose a model for the mechanism by which MSCs suppress the development of PUs in the mouse model of cutaneous I/R injury (Fig. 7). Cutaneous I/R injury causes thromboses and vascular injury, and subsequent tissue hypoxia. Hypoxia induces not only oxidative stress but also ER stress. The increased generation of ROS during oxidative stress causes apoptosis, while the increased expression of CHOP caused by ER stress may induce mitochondria-dependent apoptosis. These sequential responses may elicit PUs. The injection of MSCs may alleviate blood vessel damage and hypoxia, and subsequently suppress both oxidative stress and ER stress, resulting in the inhibition of PUs formation. Thus, the injection of MSCs can be a potential application for the early treatment of cutaneous I/R injury-induced PUs.

## Methods

### Animals

All experiments were approved by Gunma University Animal Care and Experimentation Committee (#14-066, #15-053), and carried out in accordance with the approved guidelines. C57BL/6 mice were purchased from the SLC (Shizuoka, Japan), and OKD 48 and ERAI mice were kindly provided from Dr. T. Iwawaki (Department of Life Science, Kanazawa Medical University, Ishikawa, Japan). Eight- to 12-week-old mice were used for all experiments. Mice were maintained in the Institute of Experimental Animal Research of Gunma University under specific pathogen-free conditions. Mice were handled in accordance with the animal care guidelines of Gunma University.

### Antibodies

Antibodies (Abs) and their sources were as follows: rabbit anti-mouse NG2 polyclonal Ab (pAb) (5 μg/ml; Millipore, Billerica, MA), rat anti-mouse CD31 monoclonal Ab (mAb) (5 μg/ml; MEC13.3; BD Bioscience, San Jose, CA), goat anti-8-OHdG pAb (1/200; abcam, Cambridge, UK), rabbit anti-GRP78/BiP pAb (5 μg/ml; Proteintech, Chigcago, USA), rabbit anti-CHOP/GADD153 pAb (5 μg/ml; Santa Cruz Biotechnology, Santa Cruz, CA, USA). Alexa 488- and Alexa 568-conjugated secondary Abs were obtained from Invitrogen (Carlsbad, CA).

### Cutaneous ischemia-reperfusion injury mice model

The I/R model that has been previously reported was used^5–10^. Briefly, mice were anesthetized, and hair was shaved and cleaned with 70% ethanol. The dorsal skin was gently pulled up and trapped between two round ferrite magnetic plates that had a 12-mm diameter (113 mm²) and 5 mm thick, with an average weight of 2.69 g and 1180 G magnetic forces (NeoMag Co, Ichikawa, Japan). Epidermis, dermis, subcutaneous fat layer and subcutaneous loose connective tissue layer, but not muscles, were pinched by magnetic plates. This process creates a compressive pressure of 50 mmHg between the two magnets^5^. It has been demonstrated that an external pressure of 50 mmHg is sufficient to induce skin necrosis and ulcer by reducing blood flow 80%^5^. Dorsal skin was trapped between magnetic palates for 12 hours, and then plates were removed. Mice were not immobilized, and not anesthetized during ischemia. All of the mice developed two round ulcers separated by a bridge of normal skin. For analysis, each wound sites were digitally photographed at the indicated time points after wounding, and wound areas were measured on photographs using Image J (version1.48, NIH, Bethesda, MD) as previously reported^8–10^. To assess the effects of MSCs on the development of ulcers after cutaneous I/R injury, MSC were incubated under hypoxic condition (1% O_2_, 5% CO_2_ and 94% N_2_) for 24 hours, and then, MSCs (2 × 10^6^ cells/200 μl PBS) or same volume of PBS (as a control) were injected into the dermis around the I/R area just after reperfusion (at the day of reperfusion: Day 0).

### Isolation and characterization of bone marrow-derived MSC

MSC was obtained as previously described^15,17^. The bone marrow (BM) suspension was obtained from C57BL/6 female mice between 6–10 weeks of age, and cultured in αMEM medium supplemented with 20% heat-inactivated fetal bovine serum (FBS), 2 mmol/L L-glutamine, penicillin (100 U/mL), and streptomycin (100 ug/mL). When adherent cells reached 70–90% confluence, nonadherent cells were removed, and adherent cells were harvested and expanded. Magnetic-activated cell sorting (MACS) (Miltenyi Biotec) was performed to remove CD11b+ cells according to the manufacture’s instructions. For examination of surface expression of MSC markers, BM-derived MSC was washed and incubated consecutively at 4 °C with Alexa 488-conjugated anti-human Sca-1, CD105, CD44, CD45, CD11b Ab or isotype control Ab (BioLegend, San Diego, CA) before flow cytometric analysis with a FACS Calibur instrument and CellQuest software (BD Biosciences).

### Histological examination and immunofluorescence staining

Murine skins were removed, fixed by formalin, and embedded in paraffin. Immunohistochemical staining with paraffin sections and analyses were performed as previously described^51^. Deparaffinized sections were boiled for 10 minutes for antigen retrieval. Sections were treated with endogenous peroxidase-blocking reagent (Dako) for 5 minutes and protein block (Dako) for 10 minutes at room temperature. The sections were then incubated with indicated Abs overnight at 4 °C, followed by the incubation with a horseradish peroxidase-labeled polymer-conjugated secondary Abs (ENVISION: Dako). The immunoreactivity was visualized with 3,3′-diaminobenzidine tetra- hydrochloride, and the sections were counterstained with Mayer’s hematoxylin. Immunofluorescence staining of frozen sections and analyses were performed as previously described^52^. Murine skins were removed and 4 μm frozen sections were prepared and fixed in 4% paraformaldehyde in PBS for 30 minutes. After blocking with 3% dry milk-PBS supplemented with 5% normal donkey serum or 5% normal goat serum for 1 hour at room temperature, sections were stained with Abs of interest followed by Alexa 488-, Alexa 568-conjugated secondary Abs or they were stained with Alexa 488-, Alexa 568-conjugated Ab and control proteins that were prepared using Zenon Labeling Kits (Invitrogen).Sections were counterstained with 4,6-diamidino-2-phenylindole (DAPI) to visualize nuclei, mounted in ProLong Gold antifade reagent (Life Technologies, Carlsbad, CA).

### Assessment of tissue hypoxia

Hypoxic areas after cutaneous I/R injury in I/R site were detected using the Hypoxyprobe^TM^-1 Omni kit (Hypoxyprobe, Inc., Burlington, MA) according to the manufacture’s protocol, and as described previously^9^. Pimonidazole HCl was injected intraperitoneally (60 mg/kg) 30 minutes before the sacrifice of the mice. Murine skins were removed and 4μm frozen sections were prepared and fixed in cold acetone (4 °C) for 10 minutes. Sections were incubated overnight at 4 °C with rabbit anti-pimonidazole Ab (PAb2627) diluted 1:20 in PBS containing 0.1% bovine serum albumin and 0.1% Tween 20. Sections were incubated for 1 hour with Alexa 488-conjugated secondary Ab. Images (8 fields/section) were taken and visualized with a FV10i-DOC confocal laserscanning microscope (Olympus). The positive area was determined by Image J (version1.48, NIH, Bethesda, MD) in the field (x600) as previously reported^8^.

### Apoptosis assay

The presence of apoptotic cells in the skin sections were assessed 6 days after wounding using terminal deoxynucleotide transferase dUTP nick end-labeling (TUNEL) staining kit (Roche Diagnostics, Indianapolis, IN) according to the manufacturer’s protocols, and as described previously^8,10^. Images (6 fields/section) were taken and visualized with a FV10i-DOC confocal laserscanning microscope (Olympus). The number of apoptotic cells was determined by counting TUNEL and DAPI double positive nuclei in the field (x900) as previously reported^8,10^.

### Detection of luminescent signals

Detection of luminescent signals in mice was performed as described previously^25,41,42^. Mice were sacrificed and the skin was surgically-removed and immersed in 0.3 mg/ml VivoGlo™ Luciferin, *In Vivo* Grade (Promega, Tokyo, Japan) dissolved with PBS. As soon as possible, the collect skin was placed in the *in vivo* imaging system (IVIS: PerkinElmer) imaging chamber. Date were collected with low sensitivity/30 sec exposure (ERAI mice) or high sensitivity/5 min exposure (OKD 48 mice), and analysed using LivingImage software (Xenogen).

### RNA isolation and quantitative reverse transcription-PCR

To analyze the mRNA levels of expression in I/R site by real-time RT-PCR, the whole skin samples in I/R site were used. Total RNA was isolated by RNeasy Mini Kits (Qiagen, Valencia, CA) and was subjected to reverse transcription using a SuperScript III First-Strand Synthesis System for RT-PCR (Invitrogen) according to the manufacturer’s instructions. Quantitative RT-PCR was performed with the SYBR system (Applied Biosystems, Foster City, CA) using ABI 7300 real-time PCR instrumentation (Life Technologies) according to the manufactur’s instructions. SYBR probes and primers for HO-1, NOX2, NOX4, Nrf2, Trx2, XBP-1 and GAPDH were purchased from Sigma (St. Louis, MO, USA) and Takara Bio Inc. (Otsu, Japan). As an internal control, levels of GAPDH were quantified in parallel with target genes. Normalization and fold changes were calculated using the comparative Ct method.

### ROS detection assay *in vitro*

Mouse embryonic fibroblast cells (NIH3T3) were kindly provided from Dr. S. Torii (Institute for Molecular and Cellular Regulation, Gunma University, Maebashi, Japan). NIH3T3 were maintained in Dulbecco’s modified Eagle’s medium (DMEM) containing 10% heat-inactivated fetal bovine serum (FBS), 2 mM L-glutamine, penicillin (100 U/ml), streptomycin (100 μg/ml). MSCs (5 × 10^5^ cells) were incubated in αMEM containing 1% FBS for 24 hours under hypoxic conditions, and then conditioned media were collected. NIH3T3s (2.5 × 10^4^) were cultured in OptiPlate^TM^-96F microplate (Perkin Elmer Waltham, MA). Cells were incubated in αMEN (containing 1% FBS) or MSC–conditioned medium (100 μl/well) at 37 °C for 24 hours. Cells were stimulated with 0.25 mM H_2_O_2_ (100 μl/well) for 2 hours, and then ROS levels were measured with DCFDA Cellular ROS Detection Assay Kit (abcam) according to the manufacture’s protocol and as previously reported^9,53^. Fluorescence was detected by plate reader (Perkin Elmer).

### Apoptosis and necrosis analysis by flow cytometry

Apoptosis and necrosis analysis by flow cytometry was performed as described previously^53^. MSCs (5 × 10^5^ cells) were incubated in αMEM containing 1% FBS for 24 hours under hypoxic conditions, and then conditioned media were collected. NIH3T3 cells were incubated in control medium (αMEN containing 1% FBS) or MSC-conditioned medium with or without H_2_O_2_ (0.5 mM) for 12 hours before apoptosis and necrosis analysis by flow cytometry. Both attached and non- attached cells in supernatant were corrected. Cells were treated with fluorescein isothiocyanate (FITC)-conjugated Annexin V (BD Bioscience, San Jose, CA, USA) and 7-amino-actinomycinD (7-AAD) and analysed with a FACSCalibur flow cytometer (Becton Dickinson, San Jose, CA, USA). Data were processed with FlowJo software (Tree Star Inc., Ashland, OR, USA). Cells stained positive for Annexin V and negative for 7- AAD were considered to be early apoptotic cells, and cells stained positive for both Annexin V and 7- AAD were either the end stage of apoptosis, undergoing necrosis, or already dead.

### Statistical analysis

*P* values were calculated using the Student’s *t*-test (two-sided) or by analysis of one-way ANOVA followed by Bonferroni’s post test as appropriate. Error bars represent standard errors of the mean, and numbers of experiments (n) are as indicated.

## Electronic supplementary material

Dataset 1 Supplementary Figure S1
