# Supplementary material for: Protective effect of mesenchymal stem cells on the pressure ulcer formation by the regulation of oxidative and endoplasmic reticulum stress
Source: Sci Rep. 2017 Dec 7;7:17186. doi: 10.1038/s41598-017-17630-5 (PMC5719411; doi:10.1038/s41598-017-17630-5)
Supplement: Supplementary file 1 — Dataset 1 Supplementary Figure S1 [file 41598_2017_17630_MOESM1_ESM.doc]

**Supplementary Data**

**Protective effect of mesenchymal stem cells on the pressure ulcer formation by the regulation of oxidative and endoplasmic reticulum stress**

Sei-ichiro Motegi^1^, Akiko Sekiguchi ^1^, Akihiko Uchiyama^1^, Akihito Uehara^1^, Chisako Fujiwara^1^, Sahori Yamazaki^1^, Buddhini Perera^1^, Hideharu Nakamura^2^, Sachiko Ogino^1^, Yoko Yokoyama^1^, Ryoko Akai^3^, Takao Iwawaki^3^, Osamu Ishikawa^1^

^1^Department of Dermatology, Gunma University Graduate School of Medicine, Maebashi, Japan

^2^Division of Plastic Surgery, Gunma University Graduate School of Medicine, Maebashi, Japan

^3^Division of Cell Medicine, Department of Life Science, Medical Research Institute, Kanazawa Medical University, Ishikawa, Japan

**
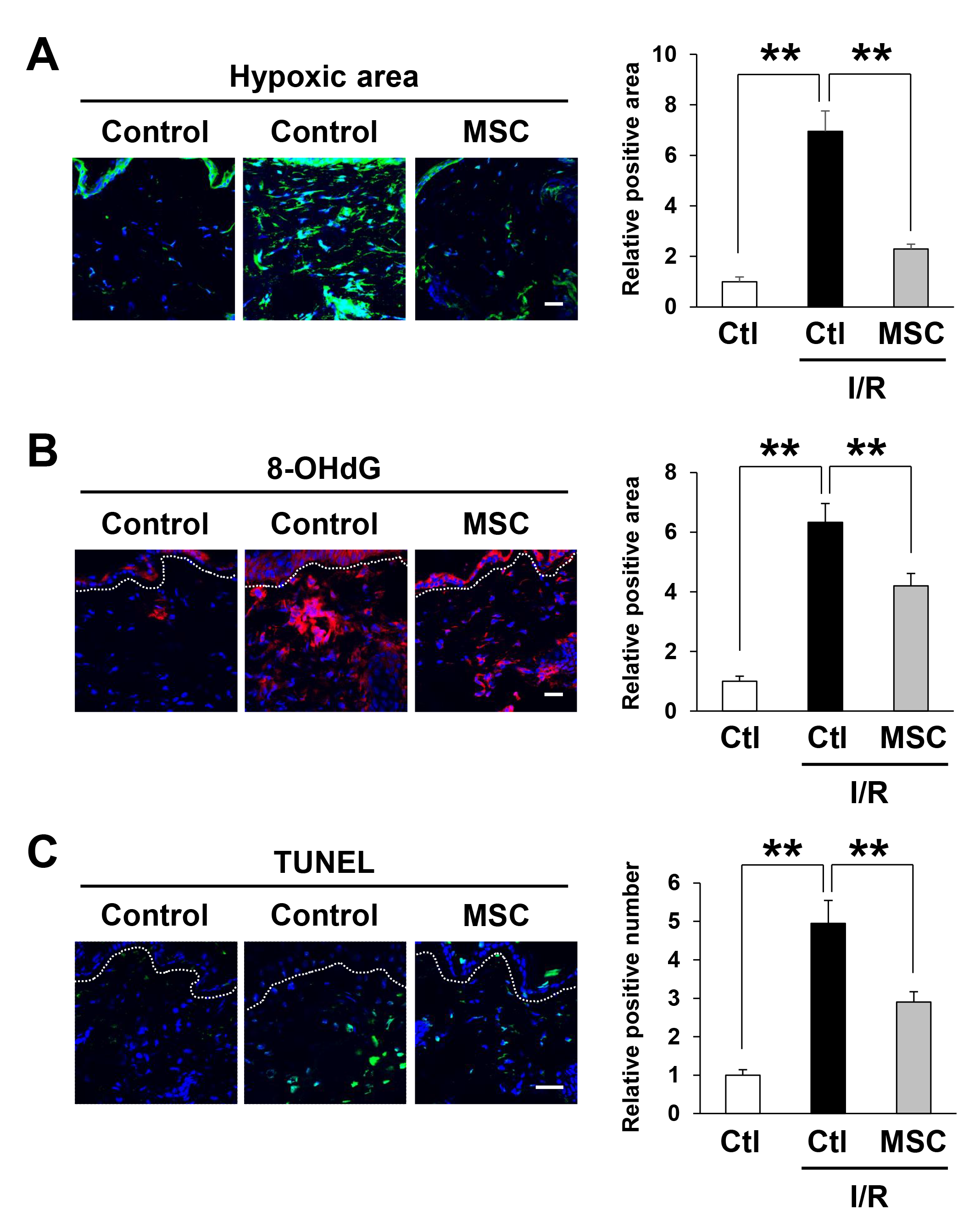
**

**Figure S1. Injection of MSCs reduced the induction of hypoxia, oxidative stress, and apoptosis by cutaneous I/R injury.** (A) The amount of pimonidazole^+^ hypoxic area (green) in cutaneous I/R site at 4 day after reperfusion. (B) The amount of 8-OHdG^+^ DNA damaged area (red) in cutaneous I/R site at 4 day after reperfusion. Quantification of the 8-OHdG^+^ areas and pimonidazole^+^ areas in 6 random microscopic fields from the center of I/R area in n=3 mice per groups was performed using Image J software. Positive area in control mice was assigned a value of 1. (C) The number of apoptotic cells in I/R site at 4 day after reperfusion was determined by counting both TUNEL and DAPI positive cells. Values were determined in 6 random microscopic fields from the center of I/R area in n=3 mice per groups. The number of apoptotic cells in control mice was assigned a value of 1. Values represent mean ± SEM. ***P*<0.01, **P*<0.05. Scale bar = 20 μm.
